# Do China’s low-carbon technology products and environmental goods trade mitigate energy-related carbon emissions in Africa?

**DOI:** 10.1371/journal.pone.0339433

**Published:** 2026-07-21

**Authors:** Dinkneh Gebre Borojo, Jiang Yushi

**Affiliations:** 1 School of Modern Business, Yiyang Vocational and Technical College, Yiyang, China; 2 School of Economics and Management, Southwest Jiaotong University, Chengdu, China; Government of Nova Scotia, CANADA

## Abstract

Motivated by the rise in green trade between China and Africa and the growing environmental pressures associated with energy-related emissions in Africa, this study examines the impacts of low-carbon technology (LT) products and environmental goods (EGs) imported from China on the energy-related carbon emissions intensity (CEI) of 42 African countries for the period 2003–2023. To account for endogeneity issues and distributional differences, the study employs instrumental variables and panel quantile techniques. The findings reveal several important points. First, LT products and EGs imported from China significantly reduce the energy-related CEI of African countries. Second, the influence of imports of LT products and EGs on energy-related CEI varies by income level: they increase energy-related CEI in low-income African economies but decrease it in middle-income African nations. Third, the import of LT products and EGs has a stronger energy-related CEI-reducing effect in African countries with better institutional frameworks. These findings contribute to the current literature by offering a detailed and nuanced understanding of how trade in LT products and EGs can help reduce energy-related carbon emissions. Policy recommendations are presented based on these findings.

## 1. Introduction

Climate change stands as one of the most pressing challenges facing societies worldwide in the twenty-first century [1]. Among these emissions, carbon dioxide (CO_2_) produced by burning fossil fuels remains the dominant source of greenhouse gases (GHG) [1,2]. Specifically, the energy sector is the dominant global contributor to GHG emissions, accounting for about 73.2%; transportation accounts for roughly 16.2%, while energy consumption in buildings accounts for around 17.5% [3]. Hence, international climate initiatives have centered on reducing energy-related CO_2_ emissions, recognizing their significant influence on global emission patterns [1].

The trend of CO_2_ emissions in Africa has been rising over the years [4], and the continent’s warming rate exceeds the global average, making it the warmest worldwide. Specifically, Africa’s high temperatures are expected to surpass current levels by 2°C in 2050, with an estimated GDP loss of around 12% [5]. To prevent these damaging impacts, emissions growth must decline sharply, and the rate of warming must be limited to 1.5 °C. In Africa, the majority of CO_2_ emissions in the energy sector originate from burning fossil fuels such as coal, oil, and natural gas for power generation or to fuel vehicles and machinery [6]. Also, over 75% of Africa’s electricity is generated from fossil fuels, posing a significant threat to the continent’s sustainable development [7]. Therefore, taking measures to reduce energy-related carbon dioxide emissions intensity (CEI) can significantly contribute to achieving emissions reduction targets. However, the main question is finding the key mechanisms for mitigating energy-related CEI in Africa.

It is argued that trade in low-carbon technology (LT) products and environmental goods (EGs) can significantly assist economies with limited domestic capacity to produce these goods in reducing CO_2_ emissions. The deployment of these products is especially critical in developing economies, including countries in Africa, which are typically not producers of these products and where their adoption can result in substantial emission reductions [8]. Therefore, transferring and applying these products will be crucial to reducing carbon emissions by promoting environmental protection and driving economic transformation [8,9]. Trade in these products, including those intended to support renewable energy, energy efficiency, energy storage, and electric vehicles, plays an important role in promoting the global shift towards climate-friendly growth. Likewise, EGs include various goods used for environmental protection, resource management and environmental monitoring [10]. They mainly comprise manufactured goods that are environmentally cleaner and more resource-efficient in their production [11]. It is also argued that meeting the carbon-reduction targets of the Paris Agreement requires deploying LT products and EGs worldwide [12].

**Fig 1:**
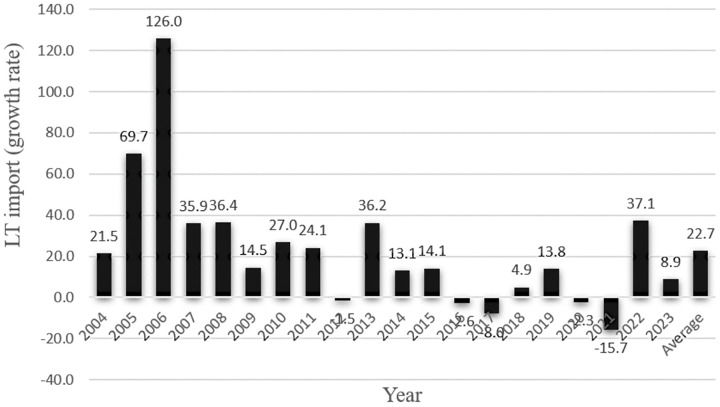
China’s LT products exports to sample African countries.

**Fig 2:**
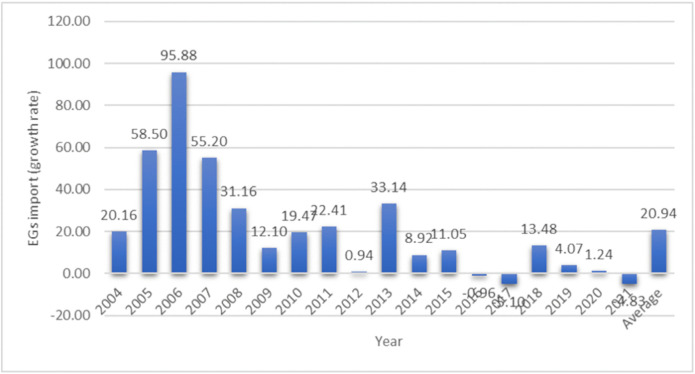
China’s EGsexports to sample African countries.

Regarding trade in LT products and EGs, China emerged as a global leader in production and exports, underscoring the shifting dynamics of the global market. China is the leading exporter of LT products globally [13]. Interestingly, China’s exports of LT products and EGs to a sample of African countries have increased significantly over the last two decades. From 2004 to 2023, China’s LT products and EGs exports to a sample of African countries increased by an average of 22.7% and 20.91%, respectively (Figs 1 and 2). Therefore, its leading role in the global low-carbon market has enabled countries worldwide, particularly those in the Global South, to access cost-effective green technologies that facilitate progress toward their environmental targets [14].

However, there are more questions about whether trade in EGs and LT products can truly promote low-CO_2_ energy development in African countries, and whether such trade is effective remains empirically untested. More specifically, a comprehensive empirical study evaluating the impacts of increasing China’s exports of LT products and EGs to African countries on energy-related CEI remains scarce, leaving an important research gap. Therefore, based on the discussions above, the main objective of this study is to assess the effects of China’s LT products and EGs exports on energy-related CEI in Africa.

From a theoretical perspective, evaluating the effects of LT products and EGs on energy-related CEI can be grounded in the technical, scale, and composition effects of trade [15]. The scale effect refers to trade in LT products, and EGs will increase the economy’s scale, thereby contributing to higher emissions [11]. The technical effect implies that trade in LT products and EGs will substitute conventional technologies and contribute to a decline in carbon emissions [11,16]. Besides, the composition effect implies that the environmental outcome of these products’ trade depends on whether this specialization occurs in energy-intensive sectors or in cleaner industries [17]. Additionally, the effects of LT products and EGs on energy-related CEI can be assessed using the STIRPAT model, a widely used framework for analyzing the human determinants of environmental change. This model typically incorporates variables such as population and per capita income (affluence), as well as other factors affecting carbon emissions [18].

From an empirical standpoint, although research on the impacts of China’s LT products and EGs exports to Africa is limited, some studies have explored the relationship between LT products, EGs trade, and carbon emissions. However, findings remain inconclusive. For example, some empirical research, such as Alvi et al. [19], Can et al. [20], Ramizo et al. [16] and Dong et al. [11], argued that EGs’ trade can lower carbon emissions. Therefore, LT products and EGs trade can play a vital role in the low-carbon transition, decarbonization and green development, and are crucial for developing economies, which are often not producers of LT products and EGs and tend to have higher emissions per unit of output than advanced economies [21,22]. On the other hand, some studies contend that EGs trade and the import of green goods do not always lead to better environmental performance and may even have little or positive effects on carbon emissions [10,17,23,24], as countries’ absorptive capacity can influence mitigation outcomes. Likewise, importing EGs may not result in measurable reductions in CO_2_ emissions [25].

Moreover, existing empirical work on the effects of LT products and EGs on decarbonization primarily focuses on a few advanced economies [20,23,26]. Also, though there are some studies on the role of the China-Africa relationship, they focus on the effects of aggregate trade on CO_2_ emissions [27], Chinese FDI on decarbonization [28], and Chinese trade and FDI on green growth [29], leaving an important research gap on the effects of LT and EG imports from China to African countries on energy-related CEI. Thus, a comprehensive analysis of China’s LT products and the impact of EGs’ exports on energy-related CEI in Africa can enrich the existing empirical literature in this area.

Therefore, this study is motivated to empirically assess the effects of China’s LT products and EGs exports on energy-related CO_2_ emissions in 42 African countries for the period 2003–2023, employing more robust econometric approaches, such as Instrumental variables-generalized method of moments (IV-GMM), method of moments quantile regression (MMQR), and Lebel’s instrumental variables (IV) strategies. Furthermore, the analysis controls for differences in institutional quality (IQ) and income levels across African countries. This is based on the premise that the effectiveness of China’s LT products and EGs exports in reducing energy-related CEI depends on recipient countries’ economic capacity and regulatory quality [30].

We focus our analysis on China’s LT products and EGs to African countries for the following reasons. First, most African countries have experienced rapid increases in CO_2_ emissions, with annual growth rates averaging 5.5% from fossil fuel sources and 6.0% from unsustainable biomass combustion [31]. Moreover, more than 65% of African countries have recorded rapid increases in consumption-based CO_2_ emissions, averaging an annual growth rate of 6.4%. Consequently, the continent’s carbon emission growth has surpassed the global average [31,32].

Second, energy consumption in Africa is rapidly increasing, a trend that indicates rising CO_2_ emissions in the region [32,33]. Therefore, despite abundant renewable energy sources, the region’s energy production still heavily depends on fossil fuels [31], thereby contributing significantly to energy-related carbon emissions. Energy demand in African countries is expected to rise sharply in the coming decades [34]. If these areas become locked into high-carbon energy systems, which are notoriously difficult to transition away from, the world could be pushed onto a higher-emissions trajectory [35].

Third, 87% of African countries are net importers of emissions, mainly due to trade relations with other developing nations, particularly in South-South trade, which accounts for 68% of all trade interactions [31]. Consequently, it is important to assess how the trade of LT products and EGs with its major trade partner (China) contributes to the greening of Africa’s energy sector.

Fourth, China has become the largest source of imports for African countries, providing LT, machinery and energy products [36,37]. China’s exports of LT products and EGs to Africa increased significantly, with an average growth of 22.7% and 20.91%, respectively, over the same period (Figs 1 and 2). Finally, we focus on imports of LT products and EGs from China, as African countries are net importers of these products.

The findings of this study make several contributions to existing work and have policy implications. First, our analysis provides novel empirical insights into how LT products and EGs imported from China affect energy-related CEI. Therefore, with the rapid development of China-Africa green products trade, exploring the relationship between China’s LT products and EGs trade with African countries and energy-related CEI can help African countries not only formulate targeted policies to achieve energy-related CEI reduction goals but also restructure their import patterns and enhance trade in LT products and EGs.

Second, unlike most existing works that focus on the broader concept of EGs, encompassing products that contribute to environmental protection and sustainable resource management, this study further delves into controlling LT product imports from China, targeting carbon emissions reductions in addition to EGs trade.

Third, this study focuses on imports of EGs and LT goods rather than total trade in EGs and LT goods, as most African countries produce a relatively small share of EGs and LT products, which are mostly imported. Additionally, unlike existing China–Africa green trade literature, which primarily focuses on aggregate trade flows or total emissions, this study examines the impact of LT products and EGs trade on energy-related CEI in Africa. Thus, by linking product-level trade data with energy-related CEI, this work can identify the mechanisms through which green technology imports from China contribute to decarbonization in Africa. Finally, this study extends the analysis by accounting for heterogeneity across African countries in terms of economic size and IQ.

Sections below provide detailed discussions on the literature review, methodology and data, findings and results, followed by discussions, conclusions, and policy implications.

## 2. Literature review

### 2.1. Theoretical background

The growing availability of LT products and EGs through trade creates significant opportunities to advance sustainable economic development [10], and they are crucial in the era of climate change and economic decarbonization. Imports of LT products denote the volume of low-carbon technology products imported by the countries. These imports encompass technologies such as wind turbines, carbon capture equipment, solar panels, and biomass systems. Aggregate imports of LT products are identified using the classification developed by Howell et al. [13], which encompasses 1245 five-digit HS codes. In general, green trade represents countries’ commitment to environmental protection and sustainable development by integrating environmentally friendly goods into trade [38].

EGs denote goods linked to environmental protection, encompassing pollution control, sustainable resource management, and products that have been adapted to be more eco-friendly [39]. As there is no globally standardized definition of EGs, various databases provide differing classifications. This study adopts the Environmental Goods Index definition and uses the IMF-identified six-digit HS codes covering both environmentally protective and environmentally enhanced goods [25]. Moreover, energy-related CEI refers to the annual CO_2_ emissions produced per unit of energy (kg/kWh). It measures total annual CO_2_ emissions, excluding those from land-use changes, expressed in kilograms of CO_2_ per kilowatt-hour of primary energy consumed.

The theoretical foundation for the analysis of the impacts of imports of LT products and EGs from China to African countries on energy-related CEI can be assessed by linking to the conceptual framework of Copeland and Taylor [15], which provides a theoretical underpinning for the channels through which trade impacts environmental outcomes. It provides evidence that trade influences environmental performance through scale, technique, and composition effects. Therefore, this framework can be a valuable lens for analyzing the relationship between LT products, EGs trade, and CO_2_ emissions intensity.

More specifically, the scale effect reflects the rise in CO_2_ emissions driven by the growth of economic activity caused by imports of LT products and EGs. In the absence of regulations, effective policies, and domestic absorptive capacity, increased imports of these goods will expand the economy’s scale, thereby placing additional pressure on the local environment and leading to higher emission levels [11]. In the case of trade in LT products and EGs, greater trade in green goods paradoxically raises emissions [17]. Therefore, if trade growth stimulates energy-intensive production, it increases the sector’s emissions, thereby raising overall carbon emissions [11].

Conversely, the technique effect refers to the impact of technological improvements on CO_2_ emissions through the importation of advanced products. When cleaner technologies are introduced, they replace older, more polluting systems, ultimately lowering carbon emissions [11], as trade can enhance availability and reduce the cost of environmentally friendly goods and technologies. This is particularly critical for economies with limited access to these goods, services, and technologies, or where domestic production capacity remains insufficient to supply them at sufficient scale or at internationally competitive prices [16]. Moreover, international trade facilitates the cross-border diffusion of environmental technologies, thereby enhancing energy efficiency and promoting environmentally sustainable production processes [40]. Also, trade-driven income growth may increase public demand for improved environmental quality, thereby mitigating emissions [16]. By facilitating greater access to goods and technologies that promote energy efficiency, trade can therefore contribute significantly to addressing environmental degradation [16].

Finally, the composition effect suggests that the environmental impact of trading these products hinges on whether a country’s specialization is concentrated in energy-intensive industries or in cleaner, low-emission sectors [17]. That is, imports of LT products and EGs can alter the allocation of economic resources across sectors with varying emission intensities, depending on a given economy’s comparative advantage [41].

### 2.2. Summary of empirical literature and hypotheses development

Building on the theoretical perspectives outlined above, numerous empirical studies have examined the effects of low-carbon and green technologies on environmental quality across economies, as well as the impact of trade in LT products and EGs on emissions. For instance, using the MMQR approach, Sharif et al. [42] examined the role of green technology in environmental quality and found that it positively influences environmental performance.

Also, employing a dynamic autoregressive distributed lag (ARDL) model, Ali et al. [39] investigated the impact of both green and non-green technological innovations on carbon emissions in Canada and indicated that green innovation significantly reduces carbon emissions. Using the MMQR approach, Ramzan et al. [43] investigated the impact of eco-friendly technologies on CO_2_ emissions across the world’s 10 most sustainable economies and found that LT significantly reduces carbon emissions.

Similarly, Fang [44] examined the effects of green technologies on CO_2_ emissions and found that LT significantly reduces pollution in China. Also, Lisha et al. [45] and Habiba et al. [46] found consistent findings for the Brazil, Russia, India, China, and South Africa (BRICS) countries and the top carbon-emitting countries, respectively. However, Sakilu and Chen [47] found that green technological innovations have a positive effect on CO_2_ emissions in the 19 countries with the highest emissions.

Furthermore, some empirical work has evaluated the effects of EGs trade on CO2 emissions, though energy-sector-specific investigations are limited. For example, Can et al. [20] investigated the role of green goods in the environmental performance of Organization for Economic Co-operation and Development (OECD) economies and confirmed that environmentally friendly goods contribute to environmental sustainability. Also, Alvi et al. [19] showed that imports of EGs in developing economies decrease emissions. Likewise, Herzer [25] implied that foreign green technology lowers CO_2_ emissions in Group-7 (G7) economies. The findings also showed that spillovers of foreign environmentally friendly technology into domestic CO_2_ emissions occur through the import of EGs.

Moreover, Dong et al. [11] argued that increased green product exports can effectively mitigate CO_2_ emissions in China. Similarly, Wei et al. [48] examined the effects of green trade and other determinants on environmental quality in the world’s top green future, and the findings showed that green trade significantly improves environmental quality.

Unlike the aforementioned empirical works, some studies argue that trade in EGs is unable to mitigate CO_2_ emissions. For instance, Bai et al. [23] analyzed the effect of green trade on CO_2_ emissions within G7 nations. Their results revealed that expanding the share of environmentally friendly trade may be counterproductive, as it contributes to higher consumption-based carbon emissions. Similarly, Hu et al. [24] suggested that trade in EGs does not necessarily yield environmental benefits in the absence of complementary policies, as the diverse end uses of such goods can increase energy consumption and, consequently, environmental degradation. Also, Liu et al. [10] and Zugravu-Soilita [17] showed that trade in EGs alone is ineffective at addressing environmental problems. Likewise, Liu et al. [49] indicated that EGs exports are detrimental to China’s green development. Based on the theoretical and empirical works discussed, the following hypothesis was developed.

***Hypothesis 1 (H1)***: *Imports of LT products and EGs from China negatively affect energy-related CO*_*2*_ *emissions in African countries.*

Moreover, it is argued that the effectiveness of LT products and EGs in mitigating carbon emissions may be constrained by recipient countries’ economic capacity and the regulatory performance of importing countries [30]. Therefore, the effects of LT products and EGs trade on CO_2_ emissions can be influenced by differences in economic development and IQ in trade partner countries. In line with this justification, Ha [50] studied the impact of EGs trade on environmental quality, distinguishing between countries with different income levels, and showed that EGs trade improves environmental quality, particularly in countries with strong and advanced institutional systems. Also, using data from 46 countries, Li et al. [51] assessed the effects of digital trade on carbon emissions, controlling for countries’ heterogeneity, and found that digital trade has a significant mitigating effect on emissions, though its impact varies across countries at different levels of development.

Moreover, IQ shapes trade composition and facilitates a shift toward cleaner and more environmentally sustainable products [52]. Thus, stable political environments and higher IQ are conducive to the adoption of LT products and EGs, as well as to the expansion of trade. Chen et al. [53] argued that IQ increases the adverse effects of green innovation on energy intensity. Thus, IQ is assumed to mitigate energy-related CEI by strengthening countries’ domestic absorptive capacity to optimize the emissions-reduction benefits of imports of LT products and EGs. Therefore, based on this foundation, the following hypothesis is articulated.

***Hypothesis 2*** *: The role of LT products and EGs from China on energy-related CEI will be influenced by income and IQ heterogeneity of African countries.*

### 2.3. Literature summary and research gaps

First, existing empirical work has investigated the effect of EGs on aggregate CO_2_ emissions and other environmental performance indicators [10,11,16,19,20,23,25,48]. However, these studies largely overlook the connection between LT products and the EGs trade and energy-related carbon emissions. Hence, investigating how China’s LT products and EGs trade affect the sectoral level, with more carbon emissions contributing to the energy sector, offers valuable insights that enrich the current body of literature on sustainable resource utilization and green finance.

Second, although empirical studies have examined the effects of EGs trade on environmental sustainability [19,23,48], they focus on developed economies or samples of emerging countries, omitting African countries and the influence of trade in LT products and EGs on energy-sector carbon emissions.

Third, prior empirical findings on the link between EGs and CO_2_ emissions are inconclusive. Some studies have reported the CO_2_-emissions-mitigation roles of EGs [11,19,25], while others have documented a positive or insignificant relationship between EGs and CO_2_ emissions [10,17,23]. Moreover, empirical work on the relationship between LT products and CO_2_ emissions from the energy sector is scarce.

Fourth, from an econometric standpoint, the findings of this study have significant methodological implications, particularly given endogeneity concerns and considerable heterogeneity. This necessitates adopting more advanced econometric approaches capable of addressing such complexities. To this end, the study employs IV-GMM and MMQR with fixed effects, which effectively address endogeneity and heterogeneity concerns.

Lastly, acknowledging the diversity among African countries in terms of IQ and income levels, this research extends beyond prior empirical works by analyzing how China’s LT products and EGs trade affect energy-related CEI in Africa while controlling for income and IQ heterogeneity. Besides, it assesses the role of regional integration in the association between imports of LT products and EGs from China and energy-related CEI in Africa. Consequently, the present study contributes a novel perspective that enriches existing research by providing more concrete evidence on how China’s LT products and EGs trade affect CEI in African countries.

## 3. Methodology and data

### 3.1. Theoretical base for model construction and data

The assessment of the relationship between the target variables (China’s LT products and EGs exports to African countries) and energy-related CO_2_ emissions intensity can be linked to Ehrlich and Holdren’s [54] and Commoner’s [55] theoretical background and the subsequent economic modeling, which has been extensively applied in the STIRPAT analysis [56]. Besides, the econometric model used in this study can be specified within the Environmental Kuznets Curve (EKC) framework, as both GDP per capita and its square are included.

Therefore, using energy-related CEI as the dependent variable, we follow a STIRPAT model and the EKC hypothesis, including per capita GDP and its square term to capture economic growth, population (PO), low-carbon technology product imports from China (LTPI), environmental goods imports from China (EGI), and other regressors. Therefore, the energy-related CEI defined in Eq (1) below is expected to be negatively affected by LTPI ($\alpha_{\text{1}}=\partial CEI_{it}/\partial LTPI_{it}<0$). Likewise, CEI in Eq (2) will be negatively influenced by EGI ($\beta_{\text{1}}=\partial CEI_{it}/\partial EGI_{it}<0$).


$$\begin{array}{c} \begin{array}{c} CEI_{it}=\alpha_{\text{0}}+\alpha_{\text{1}}LTPI_{it}+\alpha_{\text{2}}FDI_{it}+\alpha_{\text{3}}GDPpc_{it}+\alpha_{\text{4}}GDPp{c_{it}}^{\text{2}}+\alpha_{\text{5}}IND_{it}+\\ \;\alpha_{\text{6}}UR_{it}+\alpha_{\text{7}}PO_{it}+\alpha_{\text{8}}QI_{it}+\eta_{it} \end{array} \end{array}$$ (1)

Based on theoretical grounds, the *quality of institutions (QI)* and *the square of GDPpc* are expected to negatively contribute to energy-related CEI ($\alpha_{\text{3}}=\partial CEI_{it}/\partial GDPpc_{it}>0,\;\alpha_{\text{5}}=\partial CEI_{it}/\partial IND_{it}>0$). However, *GDP per capita of countries (GDPpc)*, *industrialization (IND)*, *urbanization*, and *population size* will positively influence *energy-related CEI* ($\alpha_{\text{3}}=\partial CEI_{it}/\partial GDPpc_{it}>0,\;\alpha_{\text{5}}=\partial CEI_{it}/\partial IND_{it}>0,\;\alpha_{\text{6}}=\partial CEI_{it}/\partial UR_{it}>0,\;\alpha_{\text{7}}=\partial CEI_{it}/\partial PO_{it}>0$). Finally, the effects of FDI on energy-related carbon emissions can be positive, subject to the pollution haven hypothesis ($\alpha_{\text{2}}=\partial CEI_{it}/\partial FDI_{it}>0$).


$$\begin{array}{c} \begin{array}{c} CEI_{it}=\beta_{\text{0}}+\beta_{\text{1}}EGI_{it}+\beta_{\text{2}}FDI_{it}+\beta_{\text{3}}GDPpc_{it}+\beta_{\text{4}}GDPp{c_{it}}^{\text{2}}+\beta_{\text{5}}IND_{it}+\\ \;\beta_{\text{6}}UR_{it}+\beta_{\text{7}}PO_{it}+\beta_{\text{8}}QI_{it}+\eta_{it} \end{array} \end{array}$$ (2)

### 3.2. Data

This research uses unbalanced panel data on imports of LT products and EGs from China by 42 African countries for the period 2003–2023, based on data availability for the major variables. (Sample countries: Algeria, Angola, Benin, Burkina Faso, Botswana, Burundi, Cameroon, Cabo Verde, Central African Rep., Comoros, Congo Rep., Côte d’Ivoire, Egypt, Ethiopia, Eswatini, Ghana, Gabon, Gambia, Guinea, Kenya, Lesotho, Madagascar, Malawi, Mali, Mauritius, Mauritania, Morocco, Mozambique, Namibia, Niger, Nigeria, Rwanda, Senegal, São Tomé and Príncipe, Seychelles, South Africa, Tanzania, Tunisia, Togo, Uganda, Zambia and Zimbabwe). Energy-related CEI is the dependent variable, represented by annual CO_2_ emissions per unit of energy (kg/kWh), excluding emissions from land-use change. Data on energy-related CEI are sourced from the WB database.

Besides, there are two target variables: LT products and EGs. LT products are the amount of low-carbon technology products imported from China by each sample African country, based on data from the IMF Climate Dashboard. These imports include wind turbines, solar panels, carbon capture equipment, and biomass systems [57]. The IMF adopts a top-down framework that applies a standardized methodology and uniform definition across a broad set of economies. This classification enables comprehensive assessments of aggregate bilateral trade trends in LT products. Nevertheless, because the framework does not categorize individual LT products according to specific industries or sectors, it remains limited in facilitating disaggregated sectoral analysis [58].

China’s LT products exports to African countries are the aggregate of all LT product imports from China reported by each African sample economy that meet the definition of LT products. Aggregate LT products exports are constructed by using the classification presented in Howell et al. [13]. These comprise a list of harmonized system (HS) codes (HS 2017) and are a subset of EGs [59], which includes 124 codes constituting LT products in industry supplies, capital goods, transport equipment and consumer goods [59], consisting of energy-efficient appliances, renewable energy goods, electric vehicles, energy storage solutions and smart grid systems [60].

**Fig 3:**
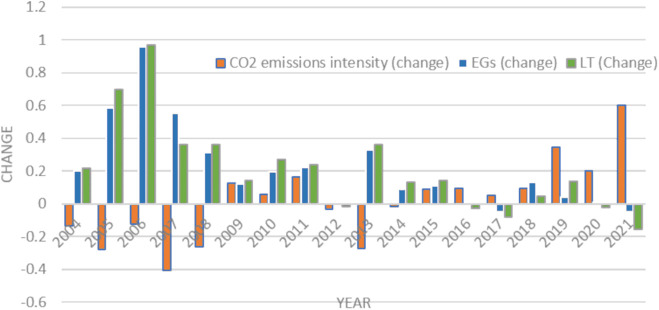
The patterns of LT products, EGs and energy-related CEI.

EGs represent imports from China to African countries, drawn from the IMF database, covering a broad range of technologies and materials that support sustainability and cleaner production practices. We use the EGs definition and data compiled by the IMF, which include goods directly associated with environmental protection, such as products used for pollution control and resource management, as well as those modified to be more environmentally friendly [59]. These goods represent an aggregation of HS 6-digit commodities classified as EGs by the International Monetary Fund (IMF) [25], including environmental protection goods across various segments, such as food and beverages, industrial and primary industrial supplies, fuels, transport equipment, capital and consumer goods [59]. The patterns in the link between the target variables and energy-related CEI are presented in Fig 3.

Moreover, based on the theoretical and empirical works, several control variables are included in the analysis. *GDP per capita, GDP per capita squared, FDI, IQ, population size, and urbanization* are included to control for economic, institutional and demographic factors that are identified as the main factors affecting CO_2_ emissions, subject to the STIRPAT framework, designed to include economic, demographic and other factors in the CO_2_ emissions analysis [18]. GDP per capita and its squared term are included to capture the EKC hypothesis, which posits that carbon emissions rise in the early stages of growth but decline as countries reach higher income levels. Population size and urbanization are used to capture the effects of demographic dynamics on energy-sector carbon emissions.

Aggregate IQ is derived from the World Bank’s (WB’s) six governance quality indicators grouped into three dimensions: economic governance, captured by government effectiveness and regulatory quality; political governance, represented by political stability and voice and accountability; and institutional governance, measured through control of corruption and the rule of law [61]. The IQ index is included in the analysis, as it is believed to affect trade composition and foster a transition towards cleaner products [52]. Principal component analysis (PCA) is applied to derive the IQ index (Table A1). The results show that institutional governance (rule of law and control of corruption) contributes more to the aggregate IQ index, followed by economic governance (government effectiveness and regulatory quality), as they have relatively larger positive loadings (Table A1). Details on the definition, data sources, and links are discussed in Table 1.

**Table 1:** Details about major variables.

Variables	Short	Definition and sources	Link
Energy-related CEI	CEI	Annual CO_2_ emissions per unit of energy (kg/kWh) represent the total yearly carbon dioxide (CO_2_) emissions, excluding those from land-use changes, measured in kilograms of CO_2_ emitted for every kilowatt-hour of primary energy consumed	https://data360.worldbank.org/en/indicator/OWID_CB_CO2_PER_UNIT_ENERGY
Imports of low-carbon technology products from China	LTPI	It represents China’s LT products exports to African countries (in dollar value per country-year pair). The data is borrowed from the IMF database	https://climatedata.imf.org/datasets/975bc577fe7342c2a3651e8841959c47_0/about
Imports of environmental goods from China by African countries	EGI	China’s EGs exports to a sample of African countries	https://climatedata.imf.org/datasets/3b7069b7bf114ebe939e3f623eb78f7b_0/explore
Real GDP per capita	GDPpc	Real GDP per capita and it is found in the World Development Indicators (WDI) database.	https://databank.worldbank.org/source/world-development-indicators
Industrialization	IND	Industry value added (% of GDP)	https://databank.worldbank.org/source/world-development-indicators
Net inflow of FDI	FDI	The net inflow of FDI and the data are borrowed from the WDI datasets.	https://databank.worldbank.org/source/world-development-indicators
Urbanization	URB	Population living in urban areas (% of total population)	https://databank.worldbank.org/source/world-development-indicators
Total population	PO	Population size of countries. The data is taken from the WDI datasets	https://databank.worldbank.org/source/world-development-indicators
Institutional quality	IQ	Aggregate IQ index derived using six governance indicators obtained from the WGI, namely control of corruption, rule of law, regulatory quality, government effectiveness, political stability, voice and accountability and absence of violence/terrorism	https://www.worldbank.org/en/publication/worldwide-governance-indicators

### 3.3. Method of analysis

An empirical model based on the EKC hypothesis and the STIRPAT framework is developed to test the role of LT products and EGs imported from China in the energy-related CEI of African countries, as shown in Eqs (1) and (2). However, there may be potential reverse causality between the target variables, the control, and the dependent variable. For example, increases in imports of LT products and EGs can influence energy-related CEI, while improvements in energy-related CEI can also affect countries’ trade performance. This bidirectional relationship may give rise to simultaneity bias, potentially yielding inconsistent and biased estimates when estimated using ordinary least squares (OLS) or standard panel regression techniques [60,62]. To address reverse causality and endogeneity concerns, the IV-GMM approach, a robust approach for controlling for potential reverse causality, is applied [63].

This approach mitigates concerns arising from endogeneity, omitted-variable bias, and measurement error, thereby ensuring more reliable and consistent results [62,63]. It also accommodates potential autocorrelation and heteroskedasticity issues. This estimator is particularly appropriate for panel datasets with a larger cross-sectional dimension than the time dimension (N = 42 > T = 21). Moreover, even in the presence of uncertain heteroskedasticity, the IV-GMM effectively mitigates omitted-variable bias, producing consistent and efficient estimates [60]. Therefore, the IV-GMM approach proposed by Baum et al. [63] is applied to estimate Eqs (3) and (4). We employ lagged values of low-carbon energy as internal instruments, given the difficulty of identifying valid external exogenous instruments [60,64]. To test the validity of the instruments, the Kleibergen-Paap rk LM statistic and the Cragg-Donald F-statistic are used to check for underidentification and weak identification, respectively, while the Hansen J test is used to check for overidentification.


$$\begin{array}{c} \begin{array}{c} CEI_{it}=\alpha_{\text{0}}+\alpha_{\text{1}}LTPI_{it}+\alpha_{\text{2}}FDI_{it}+\alpha_{\text{3}}GDPpc_{it}+\alpha_{\text{4}}GDPp{c_{it}}^{\text{2}}+\alpha_{\text{5}}IND_{it}+\\ \;\alpha_{\text{6}}UR_{it}+\alpha_{\text{7}}PO_{it}+\alpha_{\text{8}}QI_{it}+\gamma_{t}+\eta_{it} \end{array} \end{array}$$ (3)

$$\begin{array}{c} \begin{array}{c} CEI_{it}=\beta_{\text{0}}+\beta_{\text{1}}EGI_{it}+\beta_{\text{2}}FDI_{it}+\beta_{\text{3}}GDPpc_{it}+\beta_{\text{4}}GDPp{c_{it}}^{\text{2}}+\beta_{\text{5}}IND_{it}+\\ \;\beta_{\text{6}}UR_{it}+\beta_{\text{7}}PO_{it}+\beta_{\text{8}}QI_{it}+\gamma_{t}+\eta_{it} \end{array} \end{array}$$ (4)

Moreover, we used the MMQR approach for the sensitivity analysis. The MMQR explicitly captures heterogeneous effects of LT products and EGs on energy-related CEI by allowing covariates to exert varying influences across the entire conditional distribution. Accordingly, this method is well suited to analyzing data that do not follow a normal distribution (see Table A7 in S1 Appendix), as it generates robust and reliable estimates even in the presence of distributional irregularities [65]. MMQR is also resilient against outliers in panel data [42,66]. It addresses the possibility of endogenous features in the response variable [43]. Thus, we employ the MMQR to evaluate the effects of LTPI and EGI on energy-related CEI using Eqs (5) and (6).


$$\begin{array}{c} \begin{array}{c} {QCEI}_{it}\left(\tau /X_{it}\right)=\alpha_{\text{0}}+\alpha_{\text{1}}LTPI_{it}+\alpha_{\text{2}}FDI_{it}+\alpha_{\text{3}}GDPpc_{it}+\alpha_{\text{4}}GDPp{c_{it}}^{\text{2}}+\alpha_{\text{5}}IND_{it}+\\ \alpha_{\text{6}}UR_{it}+\alpha_{\text{7}}PO_{it}+\alpha_{\text{8}}QI_{it}+\varepsilon_{it} \end{array} \end{array}$$ (5)

$$\begin{array}{c} \begin{array}{c} QCEI_{it}(\tau /X_{it})=\beta_{\text{0}}+\beta_{\text{1}}EGI_{it}+\beta_{\text{2}}FDI_{it}+\beta_{\text{3}}GDPpc_{it}+\beta_{\text{4}}GDPp{c_{it}}^{\text{2}}+\beta_{\text{5}}IND_{it}+\\ \;\beta_{\text{6}}UR_{it}+\beta_{\text{7}}PO_{it}+\beta_{\text{8}}QI_{it}+\eta_{it} \end{array} \end{array}$$ (6)

Where ${QCEI}_{it}\left(\tau /X_{it}\right)$ denotes τth conditional quantile function. X set of independent variables (LTPI, EGI, FDI, GDPpc, GDPpc^2^, IND, UR, PO, and QI).

**Fig 4:**
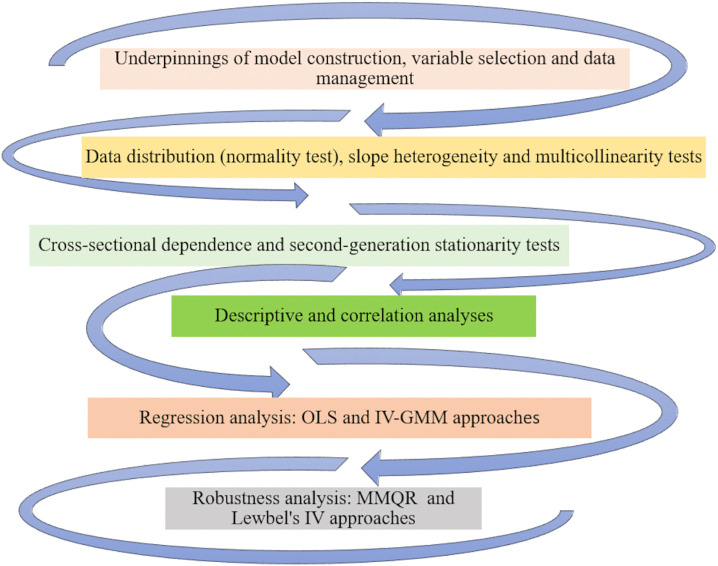
Methodological framework.


Furthermore, Lewbel’s [67] IV approach is applied to address reverse causality concerns, as it is well-suited to handle endogeneity arising from omitted-variable and simultaneity bias [28]. Therefore, external instruments are constructed for imports of LT products and EGs for each African country by using the average LT product imports and EGs share of other African countries in the sample, as specified in Eqs (7) and (8) following the work of Owusu et al. [28]. Fig 4 presents a comprehensive overview of the methods used.

$$\begin{array}{c} aveLTPI_{it}=\frac{{\text{LTPI}}_{t}-LPIT_{it}}{n-1} \end{array}$$ (7)

$$\begin{array}{c} aveEGI_{it}=\frac{{\text{EGI}}_{t}-EGI_{it}}{n-1} \end{array}$$ (8)

Where, aveLTPI_t_ and aveEGI_t_ are the average LTPI and EGI, respectively, while LTPI_t_ and EGI_t_ are the sum of LTPI and EGI from China across the sample African countries in period t, respectively, while LTPI_it_ and EGI_it_ are country-time-specific LTPI and EGI from China, and n is the total number of countries in the sample.

## 4. Results and findings

### 4.1. Preliminary analysis

Before proceeding to the regression analysis, several preliminary statistical analyses are conducted. Table 2 reports the statistical summary of the major variables included in the investigations of the effects of LTPI and EGI on energy-related CEI, followed by correlation and multicollinearity tests. The correlation coefficients between LTPI and EGI are higher (Table A2), and multicollinearity concerns arise given their individual variance inflation factor (VIF) values (Table 3A). As a result, they are separately included in the analysis. Moreover, the slope heterogeneity (S-H) test results shown in Table A4 indicate that the slope coefficients exhibit heterogeneity.

**Table 2:** Summary of major variables.

Variable	Obs	Mean	Std. dev.	Min	Max
CEI_it_	806	0.222	0.096	0.040	0.778
LTPI_it_	827	7.27E + 07	1.77E + 08	515.000	2.60E + 09
EGI_it_	757	1.06E + 08	2.47E + 08	8.000	2.30E + 09
FDI_it_	879	3.831	5.642	−17.292	56.288
GDPpc_it_	882	2365.832	2759.283	252.807	19481.6
IND_it_	849	3.890	7.963	−45.794	54.919
UR_it_	882	42.945	17.944	8.908	91.029
PO_it_	882	2.36E + 07	3.39E + 07	82475	2.30E + 08
RQ_it_	882	−0.545	0.533	−2.202	1.197

**Table 3:** The effects of LC products and EGs on CEI.

Variables	I (LT products)	II (EGs)	III (LT products)	IV (EGs)
logLT	−0.049*** (0.016)		−0.077*** (0.029)	
logGG		−0.036** (0.016)		−0.067** (0.029)
logFDI	−0.079*** (0.014)	−0.083*** (0.014)	−0.082*** (0.016)	−0.083*** (0.017)
logGDPpc	0.110*** (0.030)	0.085*** (0.031)	0.137*** (0.036)	0.113*** (0.041)
logGDPpc2	−0.011 (0.009)	−0.022** (0.009)	−0.005 (0.009)	−0.016* (0.010)
logIND	−0.008 (0.008)	−0.011 (0.008)	−0.005 (0.008)	−0.009 (0.008)
logUR	0.153*** (0.042)	0.142*** (0.043)	0.146*** (0.040)	0.130*** (0.039)
logPO	0.080*** (0.023)	0.063*** (0.022)	0.117*** (0.037)	0.103*** (0.038)
logIQ	−0.018** (0.009)	−0.009 (0.009)	−0.016* (0.009)	−0.005 (0.009)
_cons	−3.038*** (0.441)	−2.544*** (0.365)	−3.424*** (0.412)	−2.868*** (0.489)
Time fixed effects	Yes	Yes	Yes	Yes
Observations	748	720	676	649
R2	0.188	0.139	0.952	0.951
UI.(F statistic			128.837	23.97
WI. (F statistic)			219.564	182.881
Hansen J statistic			0.121	0.7863

Note: ***, **, and * show significance at the 1%, 5%, and 10% significance levels, respectively. Standard errors in parentheses. UI-under identification, WI is weak identification, and Hansen J is for overidentification.

### 4.2. Baseline results

The effects of LT products and EGs imported from China on the energy-related CEI of African countries are presented in Table 3 below. LT products and EGs are controlled separately due to the very high collinearity between them. The first two Columns (I and II) report the OLS results, and Columns (III) and (IV) present the findings of the IV-GMM approach. The diagnostic tests confirmed that the IV-GMM model is neither weakly identified, under-identified, nor over-identified. Therefore, the baseline results are interpreted using the IV-GMM findings reported in Columns III and IV.

The findings imply that GDP per capita positively affects energy-related CEI, suggesting that larger economies consume more energy and emit more carbon from the energy sector. This can be attributed to the fact that the economic growth model of most African countries has yet to transition to a low-carbon paradigm, with economic expansion continuing to generate higher carbon emissions. Moreover, the coefficient on the square term of GDP per capita is negative in all specifications. Therefore, the assumptions of the EKC framework are supported, as the coefficients for per capita GDP and its square have the expected signs. This indicates that energy-related CEI initially increases with economic expansion to a certain point, beyond which further per capita GDP growth mitigates energy-related CEI in African countries.

Moreover, FDI inflow has a significant adverse effect on energy-related CEI. Therefore, the results confirm the pollution halo hypothesis, which predicts that FDI helps reduce CO_2_ emissions by facilitating the transfer of improved management practices and environmentally friendly technologies to host countries. Likewise, the aggregate IQ indicator has a negative impact on energy-related CEI, suggesting that higher IQ levels contribute to mitigating carbon emissions from the energy sector in African countries. However, population size and urbanization positively contribute to energy-related CEI in African countries. Population size and urbanization are significant factors contributing to the increase in energy-related CEI in African countries. Urbanization increases energy emissions intensity, as urban societies typically use more electricity, appliances, vehicles, and services, which may lead to greater reliance on fossil fuels in African countries. Similarly, an increase in population size increases energy demand and results in higher emissions from energy consumption.

Regarding the target variables, the findings suggest that LT products imported from China have a significant negative effect on the energy-related CEI in African countries. Specifically, a 1% increase in China’s LT product imports to African countries reduces energy-related CEI by 0.077% (Column III, Table 3). Therefore, the results indicate that African countries’ imports from China, especially environmentally related technology products, have a significant negative impact on their energy-related CEI. These results align with both theoretical and empirical arguments. The findings can be loosely argued to show that importing LT products, such as solar panels and electric vehicles, helps reduce carbon emissions by providing access to advanced, cleaner technologies that improve energy efficiency and reduce pollution [68]. This allows countries, especially African nations, to pursue economic growth without increasing energy-sector emissions. Additionally, by promoting green technologies and low-carbon goods, imports can play a crucial role in advancing sustainable development and supporting the transition to a low-carbon economy by replacing high-emission conventional energy sources. Consequently, China’s LT product imports can help these countries meet their emissions-reduction targets under the Paris Agreement.

Similarly, EGs imports from China to African countries negatively impact the energy-related CEI of African nations. More specifically, a 1% increase in EGs imported from China reduces the energy-related CEI of African countries by 0.067% (Column IV, Table 3). This can be roughly explained by the fact that trade in EGs provides economies with more opportunities to adapt environmental technologies to local needs. These goods can help transition from fossil fuel-based energy systems to cleaner, renewable sources by enhancing energy efficiency and supporting low-carbon production in African countries’ energy sectors.

### 4.3. Robustness Analysis

First, we run the exercises excluding countries with significant carbon emissions in the sample countries, and then exclude countries with a larger proportion of LT products and EGs imported from China. Notably, South Africa, Egypt, Algeria, and Nigeria rank among the four highest emitters in Africa. In particular, South Africa is ranked 15th among the world’s largest emitters [69]. Moreover, South Africa, Nigeria, Ethiopia, and Egypt import a larger proportion of LT products from China (see Fig A1). Therefore, the analysis is repeated, excluding these countries from the sample to further control for outliers, and the results reported in Table A5 reinforce the baseline results reported in Table 3.

Second, we further examine the effects of LTPI and EGI on total CO_2_ emissions intensity (Table A6). The findings suggest that both LT products and EGs imported from China negatively affect total CO_2_ emissions intensity in African countries, indicating a role for these imports in mitigating CO_2_ emissions.

Third, the analysis is repeated using the MMQR approach to account for potential heterogeneity across the conditional distribution. Before running the MMQR strategy, a normality test is conducted to assess the distribution of the variables. The results indicate that the variables do not follow a normal distribution (Table A7). Consequently, a non-parametric approach, namely, the MMQR, is deemed appropriate for the analysis.

Regarding heterogeneity in energy-related CEI, LT products have a greater mitigating impact in African countries with higher carbon-emission intensity per unit of energy consumption. The absolute value of LT coefficients increases from 0.032 to 0.084, indicating that a 1% increase in LT product imports from China leads to a decrease in energy-related CEI by 0.032% to 0.084% across the 25^th^ to 90^th^ percentiles (Table 4). Additionally, the results clearly show that the reduction in energy-related CEI due to China’s EGs exports to African countries is substantial across the 25^th^ to 90^th^ percentiles, decreasing from 0.035% to 0.039% as the percentile increases. Therefore, the findings suggest that LT products and EGs imported from China provide greater benefits for reducing energy-related CEI in African countries with the highest proportions of energy-related CEI.

**Table 4:** The effects of LT products and EGs on energy-related CEI (MMQR).

Panel A. The effects of LT products imported from China on energy-related CEI							
**Variables**	**location**	**Scale**	**10** ^**th**^	**25** ^**th**^	**50** ^**th**^	**75** ^**th**^	**90** ^**th**^
logLT	−0.049*** (0.017)	−0.024** (0.012)	−0.010 (0.025)	−0.032* (0.018)	−0.051*** (0.017)	−0.067*** (0.020)	−0.084*** (0.026)
logFDI	−0.079*** (0.015)	0.027*** (0.010)	−0.123*** (0.027)	−0.098*** (0.019)	−0.077*** (0.015)	−0.058*** (0.014)	−0.040** (0.016)
logGDPpc	0.110*** (0.028)	−0.034* (0.019)	0.165*** (0.049)	0.134*** (0.034)	0.108*** (0.028)	0.084*** (0.028)	0.061* (0.033)
logGDPpc2	−0.011 (0.008)	0.002 (0.005)	−0.014 (0.013)	−0.013 (0.009)	−0.011 (0.008)	−0.010 (0.008)	−0.009 (0.010)
logIND	−0.008 (0.008)	−0.004 (0.005)	−0.002 (0.011)	−0.005 (0.008)	−0.008 (0.008)	−0.011 (0.009)	−0.014 (0.011)
logUR	0.153*** (0.038)	0.030 (0.027)	0.105* (0.062)	0.132*** (0.045)	0.155*** (0.038)	0.176*** (0.041)	0.196*** (0.050)
logPO	0.080*** (0.022)	0.009 (0.015)	0.066* (0.036)	0.074*** (0.026)	0.080*** (0.022)	0.087*** (0.024)	0.093*** (0.030)
logRQ	−0.018** (0.008)	0.014*** (0.005)	−0.040*** (0.012)	−0.027*** (0.009)	−0.017** (0.008)	−0.007 (0.009)	0.002 (0.011)
_cons	−3.147*** (0.317)	0.580*** (0.212)	−4.082*** (0.454)	−3.544*** (0.342)	−3.102*** (0.317)	−2.695*** (0.362)	−2.301*** (0.453)
Observations	748	748	748	748	748	748	748
**Panel II. The effects of EGs imported from China on energy-related CEI**							
**Variables**	**location**	**Scale**	**10** ^**th**^	**25** ^**th**^	**50** ^**th**^	**75** ^**th**^	**90** ^**th**^
logGG	−0.036** (0.015)	−0.002 (0.010)	−0.033 (0.021)	−0.035** (0.016)	−0.037** (0.015)	−0.038** (0.017)	−0.039* (0.020)
logFDI	−0.083*** (0.015)	0.024** (0.011)	−0.122*** (0.029)	−0.101*** (0.021)	−0.081*** (0.015)	−0.065*** (0.013)	−0.051*** (0.015)
logGDPpc	0.085*** (0.030)	−0.063*** (0.022)	0.188*** (0.054)	0.131*** (0.038)	0.078*** (0.029)	0.037 (0.029)	−0.001 (0.034)
logGDPpc2	−0.021** (0.009)	−0.002 (0.006)	−0.017 (0.015)	−0.019* (0.011)	−0.021** (0.009)	−0.023*** (0.009)	−0.024** (0.011)
logIND	−0.011 (0.008)	−0.006 (0.005)	−0.001 (0.012)	−0.007 (0.009)	−0.012 (0.008)	−0.016* (0.009)	−0.020* (0.011)
logUR	0.142*** (0.038)	0.042 (0.028)	0.073 (0.066)	0.111** (0.047)	0.146*** (0.038)	0.173*** (0.039)	0.199*** (0.047)
logPO	0.063*** (0.021)	−0.017 (0.014)	0.091*** (0.032)	0.076*** (0.024)	0.062*** (0.021)	0.051** (0.023)	0.041 (0.028)
logRQ	−0.009 (0.009)	0.016*** (0.006)	−0.035*** (0.013)	−0.021** (0.010)	−0.008 (0.009)	0.002 (0.010)	0.012 (0.012)
_cons	−2.690*** (0.371)	0.878*** (0.253)	−4.116*** (0.463)	−3.332*** (0.361)	−2.602*** (0.373)	−2.036*** (0.456)	−1.503*** (0.578)
Observations	720	720	720	720	720	720	720

Note: ***, **, and * show significance at the 1%, 5%, and 10% significance levels, respectively. Standard errors in parentheses.

Fourth, the analysis is repeated using Lewbel’s IV approach using external instruments for the target variables. The analysis outcomes are reported in Table 5 and support the results in Table 3.

**Table 5:** The effects of imports of LT products and EGs on energy-related CEI (IV).

Variables	I	II
logLTPI	−0.110*** (0.035)	
logEGI		−0.042** (0.019)
logFDI	−0.073*** (0.015)	−0.082*** (0.016)
logGDPpc	0.173*** (0.044)	0.091*** (0.035)
logGDPpc2	−0.004 (0.009)	−0.021** (0.009)
logIND	−0.006 (0.008)	−0.011 (0.008)
logUR	0.144*** (0.041)	0.140*** (0.039)
logPO	0.158*** (0.045)	0.070*** (0.026)
logIQ	−0.015* (0.009)	−0.009 (0.009)
_cons	−3.891*** (0.486)	−2.802*** (0.425)
Time fixed-effects	Yes	Yes
Observations	735	720
R2	0.947	0.948
UI.(F statistic	88.734	64.208
WI. (F statistic)	6.302	19.175
Hansen J statistic	0.315	0.2703

***, **, and * show significance at the 1%, 5%, and 10% significance levels, respectively. Standard errors in parentheses.

### 4.4. Further heterogeneity analysis

The baseline results in Table 3 indicate that imports of LT products and EGs from China negatively affect energy-related CEI in African countries. However, because the sample countries are quite diverse, these results may not apply broadly to all such economies. To address this variation, we further examine how the effects of imports of LT products and EGs from China vary across the sample countries, accounting for differences in income levels and institutional quality. First, African countries considered in the analysis are grouped into low-income and middle-income economies, and the results are presented in Table 6.

**Table 6:** The effects of imports of LT products and EGs on energy-related CEI (income variation).

Panel A. Better income		
**Variables**	**II (LT products)**	**III (EGs)**
logLTPI_it_	−0.155*** (0.036)	
logEGI_it_		−0.100*** (0.038)
Control variables	Yes	Yes
Time fixed-effects	Yes	Yes
Observations	463	437
R2	0.951	0.941
UI.(F statistic	80.901	17.346
WI. (F statistic)	157.469	118.684
Hansen J statistic	0.158	0.695
**Panel B. Low-income**		
logLTPI_it_	0.255*** (0.057)	
logEGI_it_		0.285*** (0.063)
Control variables	Yes	Yes
Time fixed-effects	Yes	Yes
Observations	212	213
R2	0.965	0.964
UI.(F statistic	30.038	35.428
WI. (F statistic)	46.513	38.035
Hansen J statistic	0.555	0.703

Note: *** indicates 1% significance level. Standard errors in parentheses. UI-under identification, WI is weak identification, and Hansen J is for overidentification.

The findings are mixed: imports of LT products and EGs from China have a strong negative impact on energy-related CEI in middle-income economies, but a positive impact in low-income African countries. A 1% increase in imports of LP products and EGs from China into middle-income countries results in decreases of 0.155% and 0.1% in energy-related CEI, respectively. Moreover, an additional analysis is conducted for middle-income countries excluding South Africa to assess whether the country’s distinctive energy structure and status as one of the world’s largest carbon emitters may disproportionately influence the aggregate results for the middle-income country sample. The results in Table A8 imply there is no considerable skewing role. Conversely, a 1% increase in imports of LT products and EGs by low-income African countries raises energy-related CEI by 0.255% and 0.285%, respectively, suggesting that greater reliance on these imports may hinder the green energy transition in these economies.

Second, the analysis is repeated, controlling for IQ variation within the sample of African countries by dividing them into groups with lower and higher IQs. The findings in Table 7 suggest that African countries with higher IQ scores experience positive effects of LT products and EGs imports from China on energy-related CEI mitigation. However, for countries with lower IQ, the effect remains positive and statistically significant, indicating that imports of LT products and EGs from China tend to exacerbate energy-related carbon emissions rather than promote a cleaner energy transition in these economies.

**Table 7:** The effects of imports of LT products and EGs on energy-related CEI (IQ variation).

Panel A. Better IQ		
**Variables**	**II (LT products)**	**III (EGs)**
logLTPI_it_	−0.215*** (0.054)	
logEGI_it_		−0.160*** (0.056)
Control variables	Yes	Yes
Time fixed-effects	Yes	Yes
Observations	298	290
R2	0.939	0.941
UI.(F statistic	50.699	66.945
WI. (F statistic)	78.961	133.77
Hansen J statistic	0.273	0.679
**Panel B. weaker IQ**		
logLTPI_it_	0.067*** (0.025)	
logEGI_it_		0.049* (0.027)
Control variables	Yes	Yes
Time fixed-effects	Yes	Yes
Observations	319	301
R2	0.9793	0.978
UI.(F statistic	52.957	8.647
WI. (F statistic)	96.867	66.222
Hansen J statistic	0.741	0.767

Note: *** and ** show significance at the 1% and 5% significance levels, respectively. Standard errors in parentheses. UI-under identification, WI is weak identification, and Hansen J is for overidentification.

## 5. Discussions

The results in Table 3 imply that LT products and EGs imported from China to African countries mitigate energy-related CEI. This implies that as African countries import more of these goods, they will gain access to advanced equipment and knowledge to upgrade domestic energy infrastructure and lower the carbon intensity of the energy sector. Additionally, the diffusion of environmental technologies through LT products and EGs trade can promote innovation, encourage green investments, and accelerate the adoption of sustainable practices in the energy sector in the trade partner African countries.

Furthermore, African countries with the largest proportion of energy-related CEI benefit more from LT products and EGs imported from China. These findings are interesting and imply that African countries with higher initial energy-related CEI have more room for improvement, as each unit of imported LT products and EGs displaces a larger volume of fossil-fuel-based emissions, thereby significantly reducing energy-related carbon emissions. Therefore, the marginal mitigating effect of LT products and EGs imported from China is stronger in African countries with high energy-related CEI.

From an empirical perspective, the findings of this study support Herzer’s [25] argument that spillovers of foreign environmental technology into nations’ CO_2_ emissions occur through imports of EGs. Also, the findings are consistent with those of Alvi et al. [19], who showed that EGs imports in developing countries can significantly reduce CO_2_ emissions. It also partly supports the arguments of Can et al. [20] that environmentally friendly products trade promotes environmental sustainability. Results further aligned with findings of Mao et al. [70], providing evidence that China’s exports of EGs facilitate carbon emission reductions and enhance the capacity of trade partner countries to achieve carbon reductions.

However, the findings of this study contrast with those of Bai et al. [23], who observed that the increasing share of green products in the country’s imports does not help reduce consumption-based carbon emissions but, in fact, significantly increases them in the G7 countries. Also, the outcome of this study contrasts with the outcomes of Zugravu-Soilita [17], who argued that trade in EGs alone fails to effectively address environmental problems across a wide range of countries.

Theoretically, the findings can be argued to support the idea that imports of LT products and EGs promote structural change in the energy sector from carbon-intensive to renewable energy sources, thereby supporting theories of sustainable growth and green innovation. Besides, theoretically, the findings of this study can be aligned with the technique effect, which describes how technological progress driven by increased imports affects CO_2_ emissions. When LT products and EGs are imported, they tend to replace traditional, high-emission methods, thereby reducing carbon emissions in the importing nation [11]. Essentially, this effect highlights how LT products and EG trade can encourage cleaner production practices and improved energy efficiency, thereby lowering carbon intensity. Therefore, by expanding access to environmentally friendly technologies and reducing their costs, LT products and EGs are vital for mitigating energy-related CEI, particularly in countries that lack large-scale or affordable domestic production capabilities.

Moreover, trade in LT products and EGs extends beyond the mere exchange of commodities, encompassing the cross-border diffusion of technological capabilities and the transmission of sustainability-oriented knowledge [71]. By facilitating the trade-driven transfer and adoption of LT products and EGs, countries can advance energy-system decarbonization while fostering industrial upgrading [72]. In summary, imports of EGs and LT products reduce energy-sector CO_2_ emissions by boosting efficiency, promoting clean energy generation, improving pollution management, enabling technology transfer, and accelerating structural transformation toward a sustainable, low-carbon energy system.

Furthermore, when African countries are segmented by income level, the effects of LT products and EGs imported from China on energy-related CEI are mixed. Interestingly, LT products and EGs imported from China mitigate energy-related CEI in middle-income African countries. Therefore, the findings reveal that the carbon-intensity mitigation effect of imports of LT products and EGs from China is stronger in middle-income African countries than in low-income economies, because middle-income countries generally have better infrastructure, institutional quality, technological absorptive capacity, and financial systems. These advantages enable more efficient adoption and use of imported green technologies, resulting in greater energy-efficiency improvements and larger reductions in energy-related CEI.

Conversely, the findings indicate that in low-income African countries, imports of LT products and EGs from China have a positive effect on energy-related CEI. This might be loosely explained by the fact that such imports can increase energy-related emissions, as they are often tied to infrastructure expansion and industrial growth, with limited capacity to absorb and use the technology efficiently. For instance, for DRC, the import of LT products can have adverse effects on green economic transitions due to limited income, and peripheral positions in trade performance will restrict access to LT products, resulting in lower effects of LT products on energy-related CEI [71]. Moreover, this outcome arises because such technologies are introduced into structurally constrained energy systems characterized by persistent fossil-fuel dependence, inadequate infrastructure, limited absorptive capacity, and rapidly expanding energy demand, all of which weaken the immediate decarbonization gains associated with imported clean technologies. Hence, the carbon-reduction benefits of green trade depend on enabling factors such as governance and better economic size [16], which most low-income African countries lack.

Additionally, the results of the study show heterogeneous effects of imports of LT products and EGs from China on energy-related CEI subject differences in IQ across African countries in the sample, with a substantial mitigating effect on energy-related CEI in countries with higher IQ. The findings align with evidence from Ha [50], which showed that the influence of trade in EGs becomes more pronounced in countries with well-developed institutional systems. Therefore, strong institutional frameworks can promote the transfer and dissemination of environmentally friendly technology products and goods trade. When institutions ensure the protection of intellectual property rights, uphold contract enforcement, and encourage innovation, trade flows are more likely to introduce and share cleaner technologies with host nations [73]. Therefore, the higher the government’s efficiency, the more pronounced the emission-reduction effect of green goods imports [74].

On the other hand, imports of LT products and EGs have an energy-related CEI-increasing effect in African countries with weak QI, on the grounds that imported LT products and EGs may be underutilized or operated in carbon-intensive energy systems that are still dominated by fossil fuels, thereby increasing emissions intensity. Hence, IQ plays a vital role in facilitating technology transfer, driving innovation that underpins sustainable development, and mitigating environmental degradation.

To sum up, this study’s evidence demonstrates that imports of Chinese LT products and EGs can accelerate the mitigation of energy-related CEI and play a substantial role in the green energy transition in Africa. Therefore, the findings provide important implications for developing economies facing challenges similar to those of African countries, such as limited technological capacity and fossil-fuel dependence, by suggesting that imports of LT products and EGs from China can accelerate low-carbon development where domestic green technology production remains weak.

## 6. Conclusions, policy implications and limitations

### 6.1. Conclusions

This study examines how China’s trade in LT products and EGs influences energy-related CEI across 42 African nations from 2003 to 2023. It employs advanced econometric techniques, including IV-GMM, MMQR, and Lewbel-IV approaches. Additionally, the analysis accounts for variations in IQ and income levels among African countries. The study findings can be summarized as follows: (1) The impact of LT products and EGs imported from China on energy-related CEI of African countries is negative and helps reduce energy-related CEI across the whole sample. (2) Regarding the distributional heterogeneity of African countries’ energy-related CEI, those with the highest energy-related CEI benefit more from LT products and EGs imported from China compared to countries with lower energy-related CEI. (3) The effects of LT products and EGs imported from China on energy-related CEI vary when the income heterogeneity of African countries is controlled. LT products and EGs imported from China help mitigate energy-related CEI in middle-income African countries and positively contribute to energy-related CEI in low-income economies. (4) The findings suggest that LT products and EGs imported from China help reduce energy-related CEI only in African countries with better QI and exacerbate energy-related CEI in African countries with weak QI.

### 6.2. Policy implications

Based on the study’s findings, the following policy implications are proposed:

First, our findings suggest that imported LT products and EGs from China help reduce energy-related CEI by supporting the inflow of green products. Therefore, policymakers should work to ease trade restrictions on LT products and EGs to encourage the diffusion of these innovations across African markets. This can be achieved through reducing tariff and non-tariff barriers to trade in LT products and EGs by applying policy measures such as lowering tariffs on green products, improving customs and border-clearance procedures, and harmonizing technical and certification standards.

Second, governments in trade partner African countries and China should develop a robust policy framework to facilitate knowledge sharing, joint ventures, and capacity-building initiatives that help recipient countries effectively use and maintain imported low-carbon technologies through LT products and EGs

Third, the heterogeneity analysis reveals that African countries with higher energy-related CEI quantiles derive greater mitigation benefits from importing LT products and EGs from China, underscoring the need for differentiated carbon mitigation strategies tailored to the carbon-intensity profiles of individual African economies. For example, highly carbon-intensive African economies such as South Africa and Nigeria should prioritize importing Chinese LT products and EGs to decarbonize the energy sector more than lower-emission economies. Therefore, these differentiated approaches would enable African economies to align green technology imports with their specific carbon-intensity profiles and development needs, thereby enhancing the effectiveness of energy-related carbon mitigation policies.

Fourth, the study’s findings underscore the critical importance of strong QI in facilitating energy-sector decarbonization through the imports of LT products and EGs from China. More specifically, in African countries with stronger QI, where imports of LT products and EGs effectively reduce energy-related CEI, governments should reinforce transparent regulatory frameworks and governance, and deepen trade with China to accelerate clean energy adoption and the diffusion of technology. In African countries with weak QI, where EGs and LT product imports may contribute to rising energy-related CEI, governments should prioritize institutional reforms and strengthen regulatory enforcement to translate these imports into meaningful carbon mitigation outcomes.

Fifth, the mixed effects of imports of LT products and EGs from China on energy-related CEI suggest the need for differentiated policy responses based on income levels to ensure gains from green trade and to effectively mitigate energy-related CEI. In particular, in middle-income economies, where LT products and EGs imports contribute to reductions in energy-sector CEI, governments should strengthen green energy policies and enhance trade partnerships with China to accelerate the diffusion of technologies and energy-efficiency gains from these imports. Conversely, in low-income countries, where such imports may inadvertently increase CEI, policymakers should prioritize institutional development, technical capacity building and supportive energy infrastructure to benefit from LT products and EGs imports.

Finally, a stronger commitment to the African Continental Free Trade Area (AfCFTA) is essential, as deeper regional integration can boost intra-African trade, reduce tariffs and non-tariff barriers, improve logistics and energy infrastructure, and lower transaction costs, thereby accelerating structural transformation. This is particularly crucial for low-income African countries, enabling them to better leverage imports of LT products and EGs from China to mitigate energy-related CEI. Moreover, governments should establish regional green value chains, alongside improved customs efficiency and regulatory coordination aligned with the AfCFTA, to further enhance the emissions-reducing potential of green imports and support a continent-wide low-carbon transition.

### 6.3. Limitations of the study

The scope of this study is limited to examining the impact of LP products and EGs imported from China to African countries on energy-related CO_2_ emissions. Future research should undertake comparative analyses of the effects of LT products and EGs trade across different regions. Although the IV-GMM and MMQR approaches employed in this study effectively address endogeneity and distributional heterogeneity, they do not explicitly capture the short- and long-run effects of imports of LT products and EGs on energy-related CEI; these effects can be evaluated separately in future studies. Also, the investigation could be expanded to assess the role of LT products and EGs trade on energy-related CEI in both developing and developed countries, given the differences in technological, economic, and emissions patterns. Finally, for low-income economies, the relationship between green goods trade and energy-related emissions should be further empirically examined to assess whether complementary conditions or rebound effects influence the overall impact. The author used ChatGPT to correct typos and improve language during manuscript preparation. Afterward, the authors carefully reviewed, edited, and approved the final version. The authors assume full responsibility for the accuracy, content, and integrity of the published work.

## Supporting information

S1 File Appendix (Tables A1-A3 & Fig A1). Tables A1-A8 and Fig A1.
(DOCX)

S2 File The original data required to replicate the manuscript’s results (EXCEL). (XLSX)

S3 File Stata syntax (dofile) used to run analysis in the manuscript. (DOCX)

## Abbreviations

ARDL: Autoregressive distributed lag
BRICS: Brazil, Russia, India, China, and South Africa
CO_2_: Carbon dioxide
EKC: Environmental Kuznets Curve
CEI: Carbon dioxide emissions intensity
EGs: Environmental goods
EGI: Environmental goods imports from China
FDI: Foreign direct investment
GDP: Gross domestic product
GDPpc: Gross domestic product per capita
G7: Group 7
GHG: Greenhouse gases
HS: Harmonized system
IQ: Institutional quality
IMF: International Monetary Fund
IND: Industrialization
IV: Instrumental variable
IV-GMM: Instrumental variable-generalized method of moments
LT: Low-carbon technology
LTPI: Low-carbon technology product imports from China
MMQR: Method of moments quantile regression
OECD: Organization for Economic Co-operation and Development
OLS: Ordinary least squares
PCA: Principal component analysis
PO: Population size; S-H, Slope-heterogeneity
STIRPAT: Stochastic impacts by regression on population, affluence, and technology
UR: Urbanization
VIF: Variance inflation factor
WB: World Bank
WDI: World Development Indicators
