# Supplementary material for: Do China’s low-carbon technology products and environmental goods trade mitigate energy-related carbon emissions in Africa?
Source: PLoS One. 2026 Jul 21;21(7):e0339433. doi: 10.1371/journal.pone.0339433 (PMC13387553; doi:10.1371/journal.pone.0339433)
Supplement: S1 File — Tables A1-A8 and Fig A1. (DOCX) [file pone.0339433.s001.docx]

**Appendix A**

**Table A1. PCA to drive IQ.**

| Eigenvalue, proportions and cumulative | | | | | | | |
| --- | --- | --- | --- | --- | --- | --- | --- |
| Component | Comp1 | Comp2 | Comp3 | Comp4 | Comp5 | Comp6 |  |
| Eigenvalue | 4.666 | 0.519 | 0.380 | 0.233 | 0.115 | 0.088 |  |
| Proportion | 0.778 | 0.087 | 0.063 | 0.039 | 0.019 | 0.015 |  |
| Cumulative | 0.778 | 0.864 | 0.928 | 0.966 | 0.985 | 1.000 |  |
| Components (eigenvectors) | | | | | | | |
| Variable | Comp1 | Comp2 | Comp3 | Comp4 | Comp5 | Comp6 | KMO |
| Control of corruption | 0.420 | -0.066 | -0.120 | -0.804 | 0.393 | 0.057 | 0.924 |
| Regulatory quality | 0.417 | -0.377 | 0.075 | 0.543 | 0.613 | 0.083 | 0.901 |
| Government effectiveness | 0.425 | -0.412 | -0.106 | 0.011 | -0.608 | 0.518 | 0.862 |
| Absence of violence and terrorism | 0.365 | 0.665 | -0.597 | 0.238 | 0.008 | 0.112 | 0.934 |
| Rule of law | 0.444 | -0.134 | -0.022 | 0.039 | -0.304 | -0.831 | 0.872 |
| Voice and accountability | 0.373 | 0.474 | 0.782 | 0.008 | -0.083 | 0.133 | 0.926 |
| Average |  |  |  |  |  |  | 0.899 |

**Table A2. Correlations among variables.**

| Variables | a | b | c | d | e | f | g | h | i | j |
| --- | --- | --- | --- | --- | --- | --- | --- | --- | --- | --- |
| logCEI_it_(a) | 1 |  |  |  |  |  |  |  |  |  |
| logLTPI_it_(b) | 0.086 | 1 |  |  |  |  |  |  |  |  |
| logEGI (c) | 0.093 | 0.967 | 1 |  |  |  |  |  |  |  |
| logFDI_it_(d) | -0.275 | -0.097 | -0.076 | 1 |  |  |  |  |  |  |
| logGDPpc_it_(e) | 0.148 | 0.152 | 0.189 | 0.045 | 1 |  |  |  |  |  |
| logGDPpc2_it_(f) | -0.198 | -0.160 | -0.168 | 0.128 | 0.098 | 1 |  |  |  |  |
| logIND_it_(g) | -0.138 | 0.076 | 0.062 | 0.112 | -0.233 | 0.024 | 1 |  |  |  |
| logUR_it_(h) | 0.213 | 0.124 | 0.142 | 0.096 | 0.665 | -0.127 | -0.266 | 1 |  |  |
| logPO_it_(i) | 0.100 | 0.731 | 0.716 | -0.211 | -0.334 | -0.329 | 0.192 | -0.199 | 1 |  |
| logRQ_it_(j) | -0.088 | -0.058 | -0.024 | 0.255 | 0.568 | 0.387 | 0.019 | 0.272 | -0.392 | 1 |

**Table A3. Multicollinearity test.**

| **Variable** | **VIF** | **1/VIF** |
| --- | --- | --- |
| logEGI | 17.000 | 0.059 |
| logLTPI | 16.430 | 0.061 |
| logPO | 4.720 | 0.212 |
| logGDPpc | 3.510 | 0.285 |
| logUR | 2.030 | 0.492 |
| IQ | 2.000 | 0.500 |
| logGDPpc2 | 1.380 | 0.723 |
| logFDI | 1.180 | 0.847 |
| logIND | 1.180 | 0.851 |
| **Mean** | **5.49** |  |

**Table A4**. **S-H results.**

|  | **(I)** |
| --- | --- |
|  | Delta |
| Delta | -5.10e+04*** |
| Adju. Delta | -4.24e+04*** |

Note: *** shows significance at 1%.

**Table A5**. The effects of LTPI and EGI on energy-related CEI (excluding outliers).

| **Variables** | **I** | **II** |
| --- | --- | --- |
| logLT | -0.074**  (0.033) |  |
| logGG |  | -0.055*  (0.030) |
| logFDI | -0.084***  (0.016) | -0.087***  (0.017) |
| logGDPpc | 0.102**  (0.045) | 0.050  (0.049) |
| logGDPpc2 | -0.010  (0.012) | -0.029**  (0.014) |
| logIND | -0.004  (0.009) | -0.009  (0.009) |
| logUR | 0.132***  (0.042) | 0.108***  (0.042) |
| logPO | 0.089**  (0.043) | 0.056  (0.042) |
| logIQ | -0.016*  (0.009) | 0.000  (0.011) |
| _cons | -2.623***  (0.565) | -1.599**  (0.668) |
| Time FE | Yes | Yes |
| Obs. | 608 | 585 |
| R2 | 0.95 | 0.951 |
| UI.(F statistic | 114.759 | 22.063 |
| WI. (F statistic) | 168.097 | 149.766 |
| Hansen J statistic | 0.117 | 0.756 |

**Table A6. The effects of LTPI and EGI on aggregate CO2 emissions intensity.**

| **Variables** | **(I)** | **(II)** |
| --- | --- | --- |
| logLT | -0.072***  (0.028) |  |
| logGG |  | -0.047*  (0.026) |
| logFDI | -0.055***  (0.013) | -0.064***  (0.014) |
| logGDPpc | 0.079**  (0.032) | 0.033  (0.035) |
| logGDPpc2 | -0.007  (0.007) | -0.013  (0.009) |
| logIND | -0.006  (0.007) | -0.010  (0.008) |
| logUR | 0.148***  (0.035) | 0.124***  (0.035) |
| logPO | 0.118***  (0.033) | 0.087**  (0.035) |
| logRQ | -0.014  (0.008) | 0.001  (0.009) |
| _cons | -3.327***  (0.335) | -2.391***  (0.483) |
| Time FE | Yes | Yes |
| Obs. | 725 | 653 |
| R2 | 0.985 | 0.989 |
| UI.(F statistic | 132.614 | 23.882 |
| WI. (F statistic) | 233.689 | 182.877 |
| Hansen J statistic | 0.141 | 0.900 |

Note: *, **, and *** indicate 10%, 5% and 1% significance levels. Standard errors in parentheses.

**Table A7. Results for the normality test.**

| **Variable** | **Jarque-Bera (Chi(2))** |
| --- | --- |
| CI | 2824*** |
| LC | 1.4e+05*** |
| EG | 1.9e+04*** |
| FDI | 1.4e+04*** |
| GDPpc | 4803*** |
| IND | 2522*** |
| UR | 22.86*** |
| PO | 6597*** |
| RQ | 51.09*** |

Note: *** indicates significance at the 1% level.

**Table A8. The effects of LT products and EG imports from China on energy-related CEI (middle-income excluding SA).**

| **Variables** | **II (LT products)** | **III (EGs)** |
| --- | --- | --- |
| logLTPI_it_ | -0.149***  (0.036) |  |
| logEGI_it_ |  | -0.090**  (0.038) |
| Control variables | Yes | Yes |
| Time FE | Yes | Yes |
| Obs. | 463 | 420 |
| R2 | 0.952 | 0.952 |
| UI.(F statistic | 80.768 | 17.652 |
| WI. (F statistic) | 149.401 | 112.531 |
| Hansen J statistic | 0.200 | 0.688 |

Note: ***p<0.01, **p<0.05. Standard errors in parentheses. UI-under identification, WI is weak identification, and Hansen J is for overidentification.
