# Supplementary material for: Do China’s low-carbon technology products and environmental goods trade mitigate energy-related carbon emissions in Africa?
Source: PLoS One. 2026 Jul 21;21(7):e0339433. doi: 10.1371/journal.pone.0339433 (PMC13387553; doi:10.1371/journal.pone.0339433)
Supplement: S3 File — (DOCX) [file pone.0339433.s003.docx]

DO FILE

*Data definition*

ECI= Energy-related CO2 emissions intensity

LTPI=China's low-carbon technology products export to African countries

EGI=China's environmental goods export to African countries

GDPpc=GDP per capita of sample countries

IND=industalization

FDI=net inflow of FDI to African countries

PO=population size

UR=urbalization

IQ=institutional quality derived from (control of corruption (cc), regulatory quality (rq), government effectiveness (ge), absence of violence and terrorism (pv), rule of law (rl) and voice and accountability (va))

*IQ derivation*

*Table A1*

pca cc ge pv rl va rq

predict pc1

pc1=IQ

*variables transformation*

natural logarithm

gen logECI=ln(ECI)

gen logLTPI=ln(LTPI)

gen logGDPpc=ln(GDPpc)

gen logIND=ln(sqrt(IND*IND+1)+IND)

gen logFDIC=ln(sqrt(FDI*FDI+1)+FDI)

gen logPO=ln(PO)

gen logUR=ln(UR)

gen logGDPpc2=(logGDPpc-mean)*(logGDPpc-mean)

*Statistical summary*

*Table 2*

sum ECI LTPI EGI FDI GDPpc IND UR PO IQ

*Correlation analysis*

*Table A2*

corr logECI logLTPI logEGI logFDI logGDPpc logGDPpc2 logIND logUR logPO IQ

*Multicollinearity test*

*Table A3*

reg logECI logLTPI logEGI logFDI logGDPpc logGDPpc2 logIND logUR logPO IQ

vif

*normaility test*

*Table A7*

jb variable name

*slope hetrogenity*

*Table A4*

xthst logECI logLTPI logFDI logGDPpc logGDPpc2 logIND logUR logPO IQ, crosssectional(logECI logLTPI logFDI logGDPpc logGDPpc2 logIND logUR logPO IQ, cr_lags(3))

*Table 3*

reg logECI logLTPI logFDI logGDPpc logGDPpc2 logIND logUR logPO IQ time*

reg logECI logEGI logFDI logGDPpc logGDPpc2 logIND logUR logPO IQ time*

ivreg2 logECI logFDI logGDPpc logGDPpc2 logIND logUR logPO IQ time* ( logLTPI =l.logLTPI l2.logLTPI ), r first gmm2s

ivreg2 logECI logFDI logGDPpc logGDPpc2 logIND logUR logPO IQ time* ( logEGI =l.logEGI l2.logEGI ), r first gmm2s

*Table 4*

mmqreg logECI logLTPI logFDI logGDPpc logGDPpc2 logIND logUR logPO IQ, abs (year) robust q(.1 .25 .5 .75 .9)

mmqreg logECI logEGI logFDI logGDPpc logGDPpc2 logIND logUR logPO IQ, abs (year) robust q(.1 .25 .5 .75 .9)

*Table 5*

ivreg2h logECI logFDI logGDPpc logGDPpc2 logIND logUR logPO IQ time* ( logLTPI= loglccrest ), r first

ivreg2h logECI logFDI logGDPpc logGDPpc2 logIND logUR logPO IQ time* ( logEGI = logenvrest ), r first

//Heterogeneity analysis

*Table 6*

*incomedummy 0=low-income, 1=middle-income (income hetrogeneity of countries)

drop if incomedummy<1

ivreg2 logECI logFDI logGDPpc logGDPpc2 logIND logUR logPO IQ time* ( logLTPI =l.logLTPI l2.logLTPI ), r first gmm2s

ivreg2 logECI logFDI logGDPpc logGDPpc2 logIND logUR logPO IQ time* ( logEGI =l.logEGI l2.logEGI ), r first gmm2s

drop if incomecategory>0

ivreg2 logECI logFDI logGDPpc logGDPpc2 logIND logUR logPO IQ time* ( logLTPI =l.logLTPI l2.logLTPI ), r first gmm2s

ivreg2 logECI logFDI logGDPpc logGDPpc2 logIND logUR logPO IQ time* ( logEGI =l.logEGI l2.logEGI ), r first gmm2s

*Table 7*

*IQdummy 0=weak IQ, 1=strong IQ (IQ hetrogeneity of countries)

drop if IQdummy<1

ivreg2 logECI logFDI logGDPpc logGDPpc2 logIND logUR logPO IQ time* ( logLTPI =l.logLTPI l2.logLTPI ), r first gmm2s

ivreg2 logECI logFDI logGDPpc logGDPpc2 logIND logUR logPO IQ time* ( logEGI =l.logEGI l2.logEGI ), r first gmm2s

drop if IQdummy>0

ivreg2 logECI logFDI logGDPpc logGDPpc2 logIND logUR logPO IQ time* ( logLTPI =l.logLTPI l2.logLTPI ), r first gmm2s

ivreg2 logECI logFDI logGDPpc logGDPpc2 logIND logUR logPO IQ time* ( logEGI =l.logEGI l2.logEGI ), r first gmm2s

*The effects of LTPI and EGI on energy-related CEI (excluding outliers)*

*Table A5*

ivreg2 logECI logFDI logGDPpc logGDPpc2 logIND logUR logPO IQ time* ( logLTPI =l.logLTPI l2.logLTPI ), r first gmm2s

ivreg2 logECI logFDI logGDPpc logGDPpc2 logIND logUR logPO IQ time* ( logEGI =l.logEGI l2.logEGI ), r first gmm2s

*The effects of LTPI and EGI on aggregate CO2 emissions intensity*

*Table A6*

ivreg2 logCO2I logFDI logGDPpc logGDPpc2 logIND logUR logPO IQ time* ( logLTPI =l.logLTPI l2.logLTPI ), r first gmm2s

ivreg2 logCO2I logFDI logGDPpc logGDPpc2 logIND logUR logPO IQ time* ( logEGI =l.logEGI l2.logEGI ), r first gmm2s

*Excluding South Africa from middle-income sample*

*Table A8*

ivreg2 logCO2I logFDI logGDPpc logGDPpc2 logIND logUR logPO IQ time* ( logLTPI =l.logLTPI l2.logLTPI ), r first gmm2s

ivreg2 logCO2I logFDI logGDPpc logGDPpc2 logIND logUR logPO IQ time* ( logEGI =l.logEGI l2.logEGI ), r first gmm2s
